# The Influence of Contextual and Theoretical Expertise on Generic and Occupation-Specific Lifting Strategy

**DOI:** 10.1177/00187208231223429

**Published:** 2024-02-01

**Authors:** Daniel P. Armstrong, Tyson A. C. Beach, Steven L. Fischer

**Affiliations:** 1 8430 University of Waterloo, Canada

**Keywords:** paramedic, spine, kinematic, kinetic, ergonomics

## Abstract

**Objective:**

To determine whether (i) low back loads and/or (ii) kinematic coordination patterns differed across theoretical expert, contextual expert and novice groups when completing both generic and occupation-specific lifts.

**Background:**

Experience has been proposed as a factor that could reduce biomechanical exposures in lifting, but the literature reports mixed effects. The inconsistent relationship between experience and exposures may be partially attributable to the broad classification of experience and experimental lifting protocols not replicating the environment where experience was gained.

**Methods:**

Purposive sampling was used to recruit 72 participants including theoretical experts (formal training on lifting mechanics), contextual experts (paramedics), and novices. Participants performed 10 barbell and crate (generic) lifts, as well as backboard and stretcher (occupation-specific) lifts while whole-body kinematics and ground reaction forces were collected. Peak low back compression and anteroposterior shear loads normalized to body mass, as well as kinematic coordination patterns, were calculated as dependent variables.

**Results:**

No significant differences in low back loads were observed across expertise groups. However, significant differences were seen in kinematic coordination patterns across expertise groups in occupation-specific lifts, but not in generic lifts.

**Conclusion:**

Increasing expertise is unlikely to minimize low back loads in lifting. However, contextual expertise did influence lifting kinematics, but only when performing occupationally specific lifts.

**Application:**

Contextual expertise may help lifters adopt lifting kinematics that enhance the tolerance of their musculoskeletal system to withstand applied loads, but does not seem to reduce the applied low back loads relative to noncontextual expert groups.

## Introduction

When developing training interventions for manual materials handling work, novices may be encouraged to adopt the movement strategies of experts, but is that a good idea? Training novice manual materials handling workers is important because evidence shows that 50% of work-related low back pain incidence occurs in the first year of employment (van Nieuwenhuyse et al., 2004). But, the likelihood of reporting low back pain also increased over a 3 year period, which the authors attributed to the inability to tolerate the cumulative loads associated with their work (van Nieuwenhuyse et al., 2004). Therefore, initial incidence within the first year may be attributed inability to tolerate the acute loading associated with a novel exposure. However, in this paper we challenge the belief that experts perform lifting in a way that minimizes their low back loads relative to novices. Noting that employees who report work-related low back pain tend to first do so in their first year of employment, it remains important to appropriately train novice workers to prevent musculoskeletal disorders (MSD). But here, we aim to first test the underlying assumption that experienced lifters use a strategy that reduces their low back loads as a preventative strategy to reduce the likelihood of developing MSD.

Adopting a movement strategy to mimic how experts move may appear logical, but the current evidence is mixed. For example, Granata et al. (1999) found that experts exhibit higher peak and variability in low back moments and loads about all three axes of motion than novice lifters, whereas Marras et al. (2006) found that experts had significantly lower average low back compression than novices. Further, experts had higher peak low back moments when fatigued in a repetitive lifting/lowering protocol (Lee et al., 2014). Differences in both peak knee angles and moments between experts and novices when lifting have been shown, with no corresponding differences in peak trunk angle or moments (Ganon et al., 1996). Finally, work by Plamondon et al. (2014) found no differences in peak low back moments between expert and novice lifters but showed that experts had lower peak lumbar flexion angles and positioned themselves closer to the load. It is clear from the literature that both kinetic (i.e., moments and loads) and kinematic (i.e., postures and motions) variables at the low back, which have been associated with risk of developing an MSD (Gallagher & Marras, 2012; Marras et al., 1993; Norman et al., 1998; Waters et al., 1993), do not consistently differ between expert and novice groups. The inconsistency in performance variables related to MSD risk calls the efficacy of emulating expert movement strategy as a proactive ergonomics strategy into question.

The conflicting results of expertise on lifting in the literature may be due, in part, to not differentiating expertise types. In general, most studies investigating the influence of expertise on lifting strategy have used a broad classification of “expertise” that usually consists of spending a minimum amount of time working in the manual materials handling sector (that is not necessarily consistent with other experts) without experiencing an injury (Granata et al., 1999; Lee & Nussbaum, 2012; Lee et al., 2014; Marras et al., 2006; Plamondon et al., 2014). Beyond not explicitly accounting for survivorship bias, this non-specific criterion does not incorporate several underlying factors that could influence movement strategy, such as theoretical knowledge of lifting mechanics. In addition to time spent completing lifting tasks there may be a need for deliberate practice, which could be informed by theoretical knowledge, to result in consistent improvements in lifting strategy to minimize MSD risk. Without considering other underlying factors that could contribute broadly to “expertise” we cannot truly understand whether or to what degree a consistent relationship exists between expertise and lifting strategy.

To overcome the limitations of previous studies which had broad definitions for expertise classification, it is important to determine how differing types of expertise influence lifting strategy. Specifically, the effect of theoretical expertise on lifting mechanics should be compared to contextual expertise as measured by time on the job. We have previously hypothesized that likelihood of consistently aiming to minimize biomechanical exposures (i.e., lower means and variability in resultant exposures) in lifting may be influenced by contextual experience on the job (Armstrong & Fischer, 2020). Research on lift training supports this possibility where lift training with augmented feedback was more effective at influencing spine motion than didactic training (Chan et al., 2022), and incorporating practical training has been suggested as a solution to improve the content and delivery of lift training programs (Denis et al., 2020). Conversely, theoretical experts likely have the knowledge on how to lower mean low back loads in lifting, but without time spent on the job they may be less likely to lift in a way to consistently minimize these resultant loads in practice. Finally, novice lifters may have higher resultant low back loads and corresponding variability as they have neither theoretical knowledge on how to lift, nor time spent lifting to inform definition of a movement objective that involves minimizing exposure.

A second important question relating to the effect of expertise on lifting strategy is whether the influence is specific to the work context in which expertise was developed, or whether it is transferable. When considering the effect of expertise on lifting strategy there may be an interaction between experience and the work sector in which it was gained to influence resultant lifting strategy (consistent with Newell (1986)) opposed to an independent effect of experience. It is therefore important to consider whether the expertise gained in the workplace is transferrable to lifting demands that differ from the specific lifting demands of their work. If for example expertise only influenced lifting mechanics specific to lifting demands present in work, then the use of generalized lifting protocols in previous studies may further contribute to the inconsistency in findings when comparing movements and spine loading variables between expert and novice lifters (Granata et al., 1999; Lee & Nussbaum, 2012; Lee et al., 2014; Marras et al., 2006; Plamondon et al., 2014). To investigate this potential confounding effect, both demanding sector specific lifts, such as stretcher and backboard lifting for paramedics (Armstrong et al., 2020), as well as generic lifting tasks should be considered when investigating the role of experience on lifting strategy.

**Figure 1.**
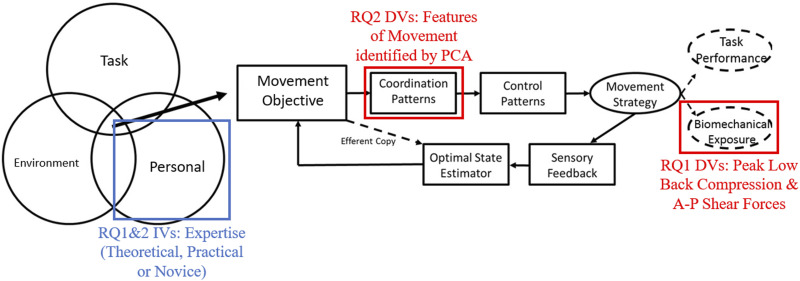
Visualization of independent and dependent variables within the study’s overarching theoretical framework. Figure adapted from Armstrong and Fischer (2020). RQ: Research Question, IV: Independent Variable, DV: Dependent Variable, PCA: Principal Component Analysis.

With a goal of investigating how experience influences lifting strategy, it is important to consider determinants of lifting strategy within the context of an overarching motor control theoretical framework. We have previously demonstrated that some individuals seem to define a movement strategy that aims to consistently minimize biomechanical exposures (i.e., lower means and variability) in their movement objectives (Armstrong & Fischer, 2020). Following these initial findings, it is prudent to next determine whether the mean and/or variability in peak low back loads (an exposure metric associated with MSD risk (Gallagher & Marras, 2012; Waters et al., 1993)) differs across expertise groups. Since low back loads are influenced by whole-body kinematics (i.e., Armstrong et al., 2021) a second important consideration is to determine whether aspects of whole-body movement strategy either associated with or not associated with low back loads differ as a function of expertise. Principal component analysis (PCA) can be applied as a method to quantify coordination patterns in movement strategy by reducing whole-body kinematic data to independent synergistic features of movement (Armstrong et al., 2019, 2021; Federolf, 2016). If low back loads are consistently lower as a function of expertise, we can probe whether independent kinematic coordination patterns associated with low back loads, as identified by PCA, are similarly controlled to achieve these lower resultant exposures. Alternatively, kinematic coordination patterns not associated with low back loads can be investigated to probe whether other potential movement objectives are adopted by experts in lifting. With a goal of testing whether expertise explains consistency and control of low back loads in lifting, the alignment of independent variables (expertise groups) and dependent variables (low back loads and kinematic coordination patterns) within an overarching theoretical framework is visualized in Figure 1.

The purpose of this study was to investigate whether type of expertise influences low back loading or corresponding movement strategy in either paramedic specific or generalized lifting tasks. To address this overarching purpose, we asked three research questions. First, across lifters classified as theoretical experts, contextual experts, and novices, are there differences in either mean or variability in peak low back compression force and anteroposterior (A-P) shear force in lifting? Second, across expertise groups are there differences in the mean or variability in synergistic features of whole-body movement (i.e., lifting strategy) that are (i) associated with low back loads or (ii) not associated with low back loads in lifting? Finally, is the effect of expertise on low back biomechanical loading or lifting strategy influenced by the type of load lifted? We hypothesized that contextual experts would have lower magnitudes of low back loads and lower variability in lifting strategy associated with low back loads suggesting they define a movement objective that involves consistently minimizing these biomechanical exposures. Second, we hypothesized that theoretical experts would have comparable low back load magnitudes to contextual experts but will demonstrate more variability in lifting strategy. Comparatively, we hypothesized that the novice group would have both higher low back loads and would exhibit more variability in lifting strategy than other groups. When considering the final research question, we hypothesized that an interaction effect would emerge between expertise and load type where contextual experts would have lower magnitudes of low back loads than theoretical experts or novices when performing paramedic lifting tasks, but not when performing the generic lifting tasks.

## Methods

### Participants

Seventy-two participants (Table 1) completed the study. A purposive sampling strategy was used to recruit working paramedics and paramedics seeking employment (contextual experts), graduate students with formal training on lifting mechanics (theoretical experts), and novice lifters (no formal experience or education in lifting). The purposive sampling approach had tightly controlled criteria to include or exclude potential participants from the study. The contextual expert groups (paramedics) must have either completed or been in the final term of a paramedic college program. Additionally, they must have been either actively completing ride outs under the supervision of paramedics performing essential work duties or be an actively employed paramedic. Theoretical experts were screened to ensure that they had at least one semester of graduate level education on biomechanics/ergonomics where they would have been taught the theoretical underpinnings of how lifting kinematics relate to low back loads. The novice group could not have any experience working in manual material handling roles, as well as not have had any relevant education in disciplines potentially related to lifting mechanics (i.e., an undergraduate degree in kinesiology). If individuals did not meet the predefined expertise group criteria (i.e., they were enrolled in their first semester of paramedic college and completed no ride outs or were an undergraduate student enrolled in a kinesiology program) they were ineligible to participate in the study. Other factors that could be related to lifting expertise, such as history of participation in strength training activities, were not considered in participant grouping. However, data collected to answer a separate research question (Armstrong et al., 2023) was used to confirm there was no significant differences in isometric lift strength or lower limb flexibility across expertise groups. This research complied with the tenets of the Declaration of Helsinki and was approved by the Institutional Review Board at the University of Waterloo. Informed consent was obtained from each participant.

**Table 1.** Participant Demographics and Number of Lifts Performed Reported as Frequency Counts, Group Means and (Standard Deviations) Stratified by Lifter Expertise Groups.

	Contextual Experts (Active Duty or In-Training Paramedics) (*n* = 20)		Theoretical Experts (*n* = 26)		Novice Lifters (*n* = 26)		Demographic Differences
	Male	Female	Male	Female	Male	Female	
Sex (*n*)	11	9	13	13	13	13	N/A
Stature (m)	1.80 (.05)	1.67 (.07)	1.77 (.07)	1.65 (.07)	1.78 (.10)	1.64 (.05)	*p* = .441,
							η^2^ = .023
Body Mass (kg)	100.8 (19.9)	81.7 (15.7)	87.5 (11.7)	70.6 (16.6)	80.3 (10.4)	71.2 (15.2)	*p* = .004*,
							η^2^ = .146
Age (yrs)	27.8 (5.5)	25.6 (3.0)	28.1 (3.8)	25.6 (3.5)	23.5 (3.0)	25.3 (2.8)	*p* = .038,
							η^2^ = .090
Barbell lifts by group (*n*)	104	88	127	128	124	129	N/A
Crate lifts by group (*n*)	109	89	120	85	123	74	N/A
Backboard lifts by group (*n*)	110	88	121	118	123	122	N/A
Stretcher lifts by group (*n*)	107	86	115	112	117	112	N/A

*Note.* Differences in demographics determined with one-way ANOVAs are reported by *p* and partial η^2^ values. Significant differences accounting for the Benjamini–Hochberg correction are denoted with “*.”

### Study protocol

Upon arrival to the lab participants were asked to complete a demographic survey and given the opportunity to familiarize themselves with all lift types. Participants were instrumented for motion capture so that whole-body kinematics could be collected during all lifting trials. To track motion, a set of 86 passive reflective markers including 46 calibration markers to define segment endpoints and 40 markers affixed to rigid bodies were attached to participants to track segmental motion. Kinematic data were collected at 100 Hz using a 12-camera passive optoelectronic system (Vicon, Oxford, UK).

**Figure 2.**
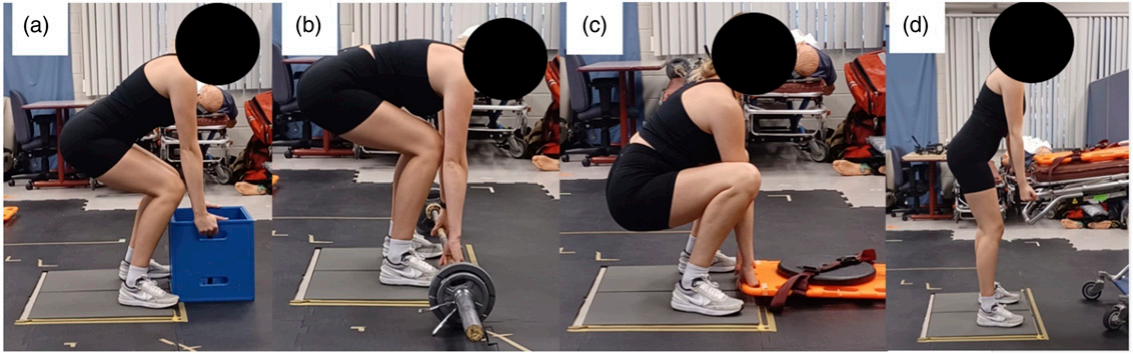
Visualization of each of the (a) crate, (b) barbell, (c) backboard, and (d) stretcher lifting conditions.

Following instrumentation, a lifting protocol was completed. During the lifting protocol participants were asked to lift each of a barbell, crate, backboard, and stretcher (Figure 2), each with an effective mass of 34 kg, for 10 repetitions in a randomized order. No instruction was provided on how to perform the lifts in an effort to elicit a self-selected lifting strategy. Backboard and stretcher lifting were included as occupation-specific lifts as they are essential tasks of paramedic work (Coffey et al., 2016) and can expose lifters to low back loads that exceed injury risk guidelines (Armstrong et al., 2020). The barbell and crate lifts were used as examples of generalized lift types, where lifting generic boxes (similar to the crate) has been previously used in research on the effect of expertise on lifting mechanics (i.e., Plamondon et al., 2014). The inclusion of occupation-relevant lifting tasks was an important consideration to have a relevant task which may elucidate differences between expert and novice groups (Farrington-Darby & Wilson, 2006). Further, the decision to use an absolute load mass with loads of the consistent proportions in the protocol was made to increase the external validity of the study where loads are not scaled to individuals’ anthropometry or strength in the workplace. Ten lifting trials to quantify lifting variability has been used previously (Granata et al., 1999) and provides relatively stable descriptions of movements performed by emergency responders (Frost, Beach, McGill, & Callaghan, 2015); this number of repetitions was decided on as a methodological trade off to expose lifters to sufficient load mass to replicate paramedic work demands while avoiding the potential for fatigue accumulation. In the barbell (lift origin height: 10 cm), crate (lift origin height: 31 cm), and backboard (lift origin height: 4 cm) lifting trials, participants were asked to lift the load from the ground to waist height, and then return the load to the ground. During the stretcher (lift origin height: 57 cm) lifting trials, participants were asked to lift and load the stretcher into a mock ambulance. Rest times of 1–2 minutes after every lift, and 3–5 minutes after every 5 lifts were taken to minimize the likelihood of fatigue accumulation. As lifting a backboard and stretcher is a two-person task, a trained lifting partner who remained consistent across participants lifted the opposite end of the equipment. During all lifting trials, participants stood with each foot on individual ground embedded force platforms (Bertec Corporation, Columbus, OH, USA). Ground reaction forces were spatially and temporally synchronized with kinematic data and collected at 1000 Hz. Based on participants’ fitness and comfort levels a subset of 63 participants performed the crate lifts, 71 completed the backboard lifts, 70 completed the stretcher lifts, and all 72 participants performed the barbell lifts.

### Data Conditioning and Preprocessing

Kinematic data were inspected for mislabeling and gaps in Vicon Nexus 2.6 (Vicon, Oxford, UK) software. Gaps less than 200 ms in duration were filled using a cubic spline, while gaps greater than 200 ms were filled using a pattern or rigid body fill, consistent with best practice (Howarth & Callaghan, 2010). For all lifting trials both the approach to the load and lifting the load from the ground to waist height were included in analysis. The start and end points of lifting trials were defined as the time point when hand linear velocity in the vertical direction was zero before approaching the load and after lifting the load to waist height.

Kinematic and ground reaction force data were imported into Visual3D software (CMotion, Germantown, MA, USA). Both kinematic and force data were filtered using a second-order dual-pass Butterworth filter with an effective cut-off frequency of 6 Hz (Winter, 2009). The filtered kinematic data were used to drive a bottom-up inverse dynamics model with anthropometrics defined based on default settings in Visual3D.

### Data Processing and Analysis

To calculate low back compression and A-P shear forces at the low back time-series low back flexion/extension angles and moments (calculated following best practice (Wu et al., 2002, 2005)) were exported to Matlab (The Mathworks, Natick, USA). To estimate muscular contributions to resultant low back compression and A-P shear forces a single muscle equivalent model was used. Since the moment arm and orientation of the lumped extensor muscles changes with posture, we used a polynomial equation (van Dieën & de Looze, 1999) to estimate the lumped extensor muscle moment arm and line of action as a function of the low back flexion/extension angle. Differences in body size and morphology on muscle moment arm were not considered in this modeling approach. Peak compression and A-P shear within each trial were extracted and then normalized to participant body mass to account for body mass differences across expertise groups (Table 1). The appropriateness of normalizing resultant loads was confirmed by testing the intercept, correlation, and statistical difference assumptions as previously recommended (Hirsch et al., 2022). The mean and within participant standard deviation of the peak compression and A-P shear loads were calculated for each lift condition to serve as dependent variables to answer the first research question.

**Figure 3.**
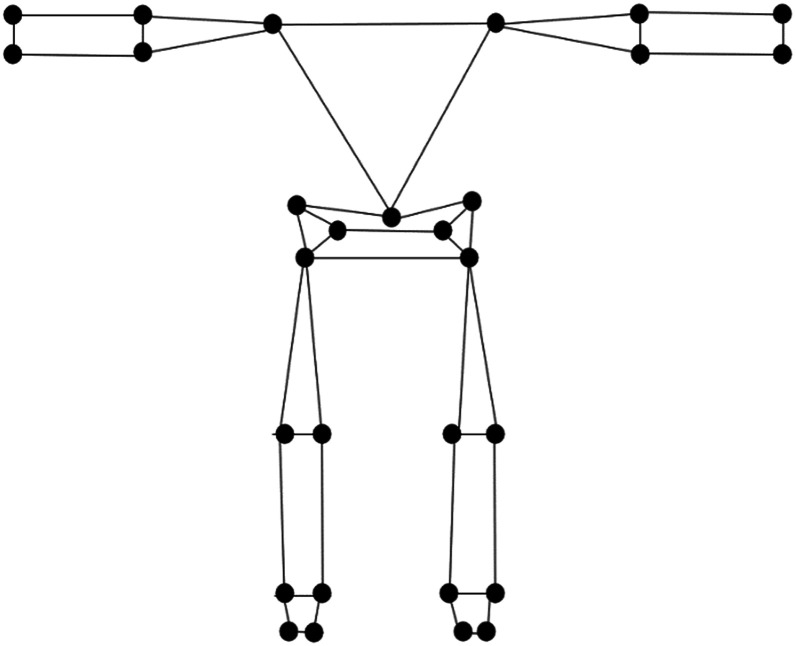
Visualization of the body landmarks for which the time-series *x, y, z* trajectories were input to the lift-specific PCA models.

To quantify lifting strategy, *x, y, z* trajectory data of 29 body landmarks (Figure 3) were exported from Visual3D to measure whole-body movement. Principal component analysis (PCA) was applied to the kinematic trajectory data to identify synergistic features of whole-body movement strategy (i.e., principal components (PCs)) consistent with previously reported methods (Armstrong et al., 2019, 2021; Remedios et al., 2020; Ross et al., 2018). All landmark trajectory data were expressed in the global reference frame and normalized in the amplitude domain to participant stature. To represent lifting strategy across a trial, posture vectors from each of the 101 timepoints were concatenated to generate a single vector to represent whole-body movement trajectory over the time series where each trial was input as an independent row vector to the PCA model. Separate PCA models were applied to independently identify features of whole-body movement in each of the four lift types. PCs explaining 90% of variance in the data set were retained for analysis (Jackson, 1991). The mean and within participant standard deviation of PC scores for all retained PCs were calculated for each lift condition to serve as dependent variables to answer the second research question.

Prior to hypothesis testing simple regression was first used to identify what PCs were associated with either peak low back compression or A-P shear forces in each lift type. Given the purpose of this study was to examine how expertise influenced low back loads in lifting, it was important to differentiate PCs associated with low back loads from those that were not.

### Hypothesis Testing

To determine the influence of expertise on low back loads across the four lift types, two-way mixed ANOVAs (α = 0.05) with a between factor of expertise group, and a within factor of lift type were used. Since PCA was applied separately to kinematic trajectory data for each of the four lift types, one-way ANOVAs (α = 0.05) were used to test for differences in PC scores in each lift between expertise groups. All statistical analyses were conducted using SPSS v. 25 (IBM SPSS Statistics), using an alpha level set at *p* < 0.05. A Benjamini–Hochberg correction (Benjamini & Hochberg, 1995) was applied to control for family-wise error across multiple ANOVAs with a false discovery rate set to 10%. An “*” is used to denote significance in through the results accounting for the Benjamini–Hochberg correction.

If PC scores were found to differ as a function of expertise than single component reconstruction (Brandon et al., 2013) and consideration of the landmark-specific loading vector (Armstrong et al., 2021) were used to determine the mode of variance explained by the differing feature of movement. Gross movement differences across expertise groups were also visualized using aggregate component reconstruction using previously reported methods (Armstrong et al., 2021). In the aggregate component reconstruction loading vectors of all individual PCs that differed as a function of expertise groups were multiplied by the group-specific mean PC score and then added to the mean whole-body movement trajectory. The use of a mean trajectory in the reconstruction assumes a normal distribution of kinematic strategies. However, with a large sample of trials used in the study we do not anticipate that potential nonnormality in our data largely influenced the identified mean trajectory. Further, outliers in input kinematics are unlikely to be represented in retained PCs explaining higher proportions of variance, and are therefore unlikely to reduce the biological realism of the aggregate component reconstructions. Post-hoc testing was used to confirm expertise groups differed from one another prior to multiplying loading vectors by group mean PC scores. When no significant post-hoc differences between two expertise groups were observed, the loading vectors were multiplied by the mean PC score magnitude between the two groups.

## Results

No significant differences in the mean or variability of peak low back compression or shear loads were observed across expertise groups (Table 2). However, the mean peak compression and A-P shear forces, as well as variability in A-P shear force differed as a function of lift type. No significant interaction effects were observed.

**Table 2.** Differences in Mean and Variability of Peak Low Back Compression and Anteroposterior Shear Forces Normalized to Body Mass Across all Lift Types and Between Expertise Groups.

	Expertise			Lift Types				Statistical Summary		
	Novice	Context.	Theo.	Bar.	Back.	Crate	Stretch.	Expertise	Lift	Interact.
Mean comp. (N/kg)	50.24 (14.14)	47.21 (16.41)	48.34 (13.37)	55.66 (15.52)	54.11 (15.60)	55.04 (14.44)	28.32 (11.56)	*p* = .669, η^2^ = .013	*p* < .001*, η^2^ = .775	*p* = .331, η^2^ = .037
Mean shear (N/kg)	7.08 (3.54)	6.98 (3.33)	6.60 (3.62)	9.55 (5.40)	9.47 (5.87)	6.03 (2.04)	2.76 (1.18)	*p* = .955, η^2^ = .002	*p* < .001*, η^2^ = .523	*p* = .979, η^2^ = .006
Stdev. Comp. (N/kg)	5.26 (2.38)	3.89 (2.07)	4.55 (2.60)	4.08 (2.26)	4.69 (2.52)	4.67 (2.50)	4.86 (2.34)	*p* = .019, η^2^ = .125	*p* = .200, η^2^ = .026	*p* = .117, η^2^ = .055
Stdev. Shear (N/kg)	1.54 (1.77)	1.85 (3.49)	1.42 (1.66)	1.77 (1.67)	3.24 (7.39)	.85 (.72)	.59 (.42)	*p* = .816, η^2^ = .007	*p* < .001*, η^2^ = .105	*p* = .740, η^2^ = .019

*Note.* Significant differences accounting for the Benjamini–Hochberg correction are denoted with “*.” Comp: Compression, Stdev: Standard Deviation, Context: Contextual, Theo: Theoretical, Bar: Barbell, Back: Backboard, Stretch: Stretcher.

Across lift types between 14–20 PCs were retained for analysis, with nearly half of those PCs being associated with either peak low back compression and/or A-P shear loads (Table 3). PCs associated with resultant peak compression or A-P shear loads are referred to as associated with low back loads.

**Table 3.** Variance Explained (%) by PCs in Each of the Barbell, Backboard and Crate Lifting Conditions.

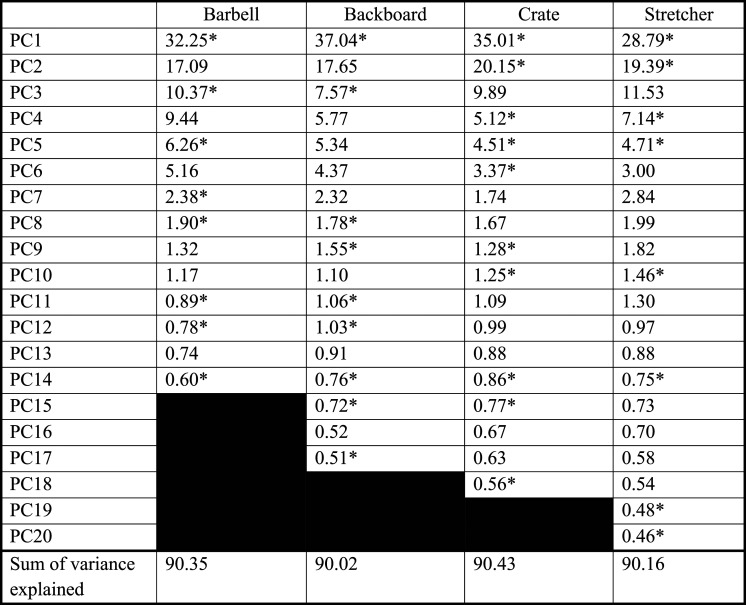

*Note.* An “*” indicates that the PC scores within a given PC were associated with either peak low back compression or A-P shear force.

While no consistent differences in the means or variability of peak low back loads were seen across expertise groups, there were differences in both backboard and stretcher lifting strategy in both PCs associated with low back loads, and not associated with low back loads (Table 4).

**Table 4.** Statistical Summary (*p*-Values and Partial η^2^) Indicating Whether the Mean or Standard Deviation of Principal Component (PC) Scores Differed as a Function of Expertise Group.

Lift	Associated with Low Back Loads			Not Associated with Low Back Loads		
	PC	*p-*values, Partial η^2^		PC	*p*-values, Partial η^2^	
		Mean Scores	Stdev. Scores		Mean Scores	Stdev. Scores
Barbell	PC1	.148, .054	.056, .080	PC2	.084, .069	.408, .026
	PC3	.996, .000	.267, .038	PC4	.932, .002	.758, .008
	PC5	.779, .007	.632, .013	PC6	.011, .122	.598, .015
	PC7	.114, .061	.953, .001	PC9	.109, .062	.678, .011
	PC8	.232, .042	.461, .022	PC10	.080, .070	.247, .040
	PC11	.997, .000	.399, .026	PC13	.625, .014	.418, .025
	PC12	.636, .013	.398, .026			
	PC14	.433, .024	.010, .126			
Backboard	PC1	.227, .042	.457, .023	PC2	.006*, .141	.192, .047
	PC3	.001*, .180	.349, .031	PC4	.339, .031	.028, .100
	PC8	.068, .076	.100, .065	PC5	.553, .017	.135, .057
	PC9	.129, .058	.197, .047	PC6	.060, .079	.180, .049
	PC11	.085, .070	.660, .012	PC7	.738, .009	.056, .081
	PC12	.027, .101	.339, .031	PC10	.090, .068	.237, .041
	PC14	.344, .031	.355, .30	PC13	.221, .043	.034, .094
	PC15	.342, .031	.074, .074	PC16	.192, .047	.041, .090
	PC17	.023, .105	.109, .063			
Crate	PC1	.910, .003	.437, .027	PC3	.286, .040	.297, .040
	PC2	.573, .018	.172, .056	PC7	.305, .038	.326, .037
	PC4	.066, .084	.010, .142	PC8	.063, .085	.109, .071
	PC5	.055, .089	.221, .049	PC11	.615, .016	.489, .024
	PC6	.419, .028	.717, .011	PC12	.303, .038	.098, .075
	PC9	.683, .012	.185, .055	PC13	.409, .028	.094, .076
	PC10	.133, .063	.004*, .169	PC16	.792, .007	.079, .081
	PC14	.358, .033	.425, .028	PC17	.915, .003	.207, .051
	PC15	.391, .030	.340, .035			
	PC18	.421, .028	.878, .004			
Stretcher	PC1	<.001*, .299	.023, .109	PC3	.619, .015	.759, .008
	PC2	.949, .002	.963, .001	PC6	.105, .067	.845, .005
	PC4	<.001*, .281	.268, .040	PC7	.899, .003	.082, .074
	PC5	.097, .069	.437, .025	PC8	.759, .008	.206, .047
	PC10	.588, .016	.150, .057	PC9	.523, .020	.500, .021
	PC14	.932, .002	.616, .015	PC11	.089, .072	.085, .073
	PC19	.645, .013	.151, .056	PC12	.863, .005	.095, .070
	PC20	.138, .059	.286, .038	PC13	.005*, .148	.220, .046
				PC15	.318, .035	.161, .055
				PC16	.836, .006	.004*, .157
				PC17	.766, .008	.038, .096
				PC18	.520, .020	.291, .037

*Note.* Significant differences accounting for the Benjamini–Hochberg correction are denoted with ‘*’.

**Figure 4.**
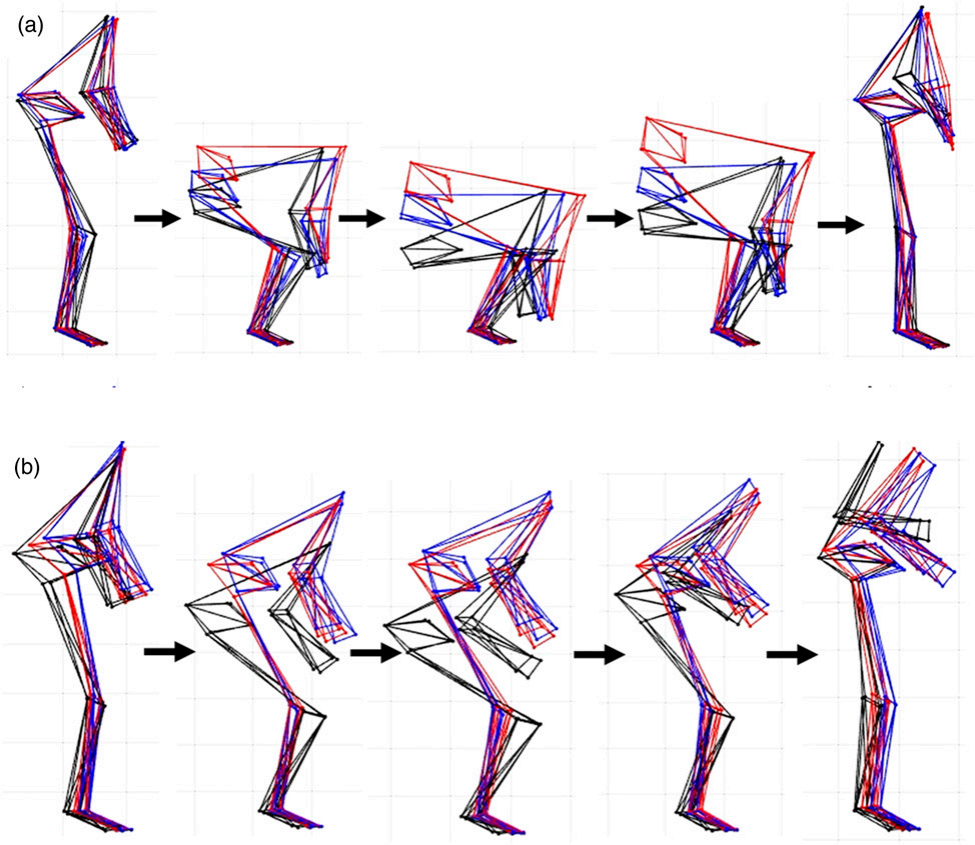
Aggregate component reconstructions of (a) backboard and (b) stretcher lifting across contextual experts (black), theoretical experts (blue) and novice (red) lifters. When lifting a backboard, contextual experts used more knee bend, resulting in a lift initiation postures where their head was above their hips (more upright), relative to theoretical experts and novices. Though the lift origin was higher for the stretcher lift, contextual experts continued to use more knee bend, resulting in a lower pelvis position to maintain a more upright spine.

Based on the statistical differences in PC scores in backboard and stretcher lifting strategy, the gross kinematic strategy adopted by expertise groups is visualized by aggregate component reconstructions (Figure 4). Interpreting these differences in backboard lifting the contextual expertise group (paramedics—black line) adopted a deeper squat while maintaining a more upright trunk in the global reference frame (Figure 4(a)). In contrast the novices (red line) adopted a more stoop-like lift posture with greater trunk flexion. The theoretical expert reconstruction falls between these two lift postures. In the stretcher lift the paramedics seem to move their body through the greatest range of motion by adopting a deeper squat to initiate the lift, and then lifting the stretcher to higher in the vertical (Figure 4(b)).

## Discussion

The purpose of this study was to investigate whether expertise is a determinant of lifting strategy in either paramedic specific or generalized lifting tasks. We found that the means and variability of peak low back compression and A-P shear force generally did not differ across expertise groups, but they did differ as a function of lift type. While no differences in peak low back loads were observed between expertise groups there were differences in both backboard and stretcher lifting strategy (occupationally relevant lifting tasks), with no differences in mean lifting strategy in either barbell or crate (generic) lifting tasks. These findings highlight that experts do not seem to define a movement objective that aims to consistently minimize low back loads in lifting. However, expertise did influence the lifting strategy used in lifting tasks specific to their occupation. While contextual expertise did not relate to a decrease in low back loads, it is possible that their lifting strategy resulted in postures that may be better to tolerate the exposures as demonstrated by practical experts having a more upright trunk in the aggregate component reconstructions (Figure 4). Practically, these results suggest that simply emulating expert lifting strategy as a proactive ergonomic intervention to reduce MSD risk is unlikely to reduce joint load exposures to the body but may help novices adopt postures that can better tolerate those exposures. The important caveat is that contextual experts only demonstrated postures that may better tolerate low back loads when perform job-specific lifts, suggesting that generic training programs may not transfer to occupationally specific lifting.

Our hypothesis that contextual experts would consistently minimize low back loads in lifting was not supported. This hypothesis was primarily motivated by epidemiological evidence which demonstrates that the development of work-related low back pain is greater in inexperienced worker groups (van Nieuwenhuyse et al., 2004), and that augmented feedback while performing lifting tasks more greatly influenced spine motion compared to instructional training alone (Chan et al., 2022). However, the mechanism for this relative difference in MSD incidence between novice and experienced workers does not seem attributable to experts consistently moving in a manner that would reduce their corresponding peak low back loads. Further, theoretical knowledge on lifting mechanics was also not shown to result in consistent reductions low back loads. Previous literature has highlighted that theoretical lifting experts have preferences on lifting technique (Abdoli-Eramaki et al., 2019), but the results of this study demonstrate that these underlying beliefs do not translate into practice contrary to our hypothesis. Although the view that experts have lower resultant loading is a logical and pervasive belief, the results of this study importantly support that expertise as defined in this study is not a causative factor that results in reduced low back loads in lifting. We suggest that experts may be better able to tolerate load, where “safe lifting” programs might be better aligned to help novice lifters develop the tolerance to lifting. Hypothetically, this may be achieved via management of external (i.e., “amount” of lifting) and internal (i.e., lifting “technique”) loads together with general physical preparation training (i.e., exercise).

Though no differences in the mean or variability of low back loads were observed as a function of expertise, we did observe differences in backboard and stretcher lifting strategy. Trained paramedics adopted deeper squat postures to initiate both the backboard and stretcher lifting tasks. Correspondingly they also had less trunk flexion at lift initiation compared to the theoretical expert and novice groups. Interestingly, these differences in lift postures did not result in differences in peak low back loads across groups which is likely due to all groups initiating the lift through leg extension, followed by trunk extension. The noted leg then trunk extensions sequential lifting strategy is thought to minimize the peak low back extensor moment by delaying back extension until after 25% of the lift cycle where acceleration of the load is greatest (De Looze et al., 1993). One potential reason for the differences in lifting strategy may be that groups aimed to achieve differing implicit and explicit movement objectives. For example, by adopting a more upright posture, paramedics were able to better preserve eye contact with their lifting partner when lifting the backboard. Communication between lifting partners is an important feature of safe lifting for paramedics and many noted (anecdotally) that eye contact was a critical component of lifting. Theoretical or novice lifters may not have understood the importance of eye contact, and thus did not move in a way the preserved line of sight to their lifting partner in the same way. This finding reinforces the importance of contextual factors, like the need to see and communicate with a lifting partner, which can play an important role in dictating the corresponding lifting strategy.

While no differences in low back loads were observed across expertise groups, kinematic differences during occupation-specific lifts may influence underlying tissue tolerance as a strategy to reduce MSD risk. Notably, in the backboard lifting task experts maintained a more upright trunk and used motion about their lower extremities to a greater extent when performing the lift (Figure 4). Maintaining a more upright trunk posture could increase tissue tolerance at the low back as in vitro testing has shown that functional spinal unit specimens have a lower yield point and compressive strength when in a flexed compared to neutral posture (Gunning et al., 2001). Further, the upright trunk posture adopted by theoretical experts may contribute to increasing tissue tolerance as posture/motion modulates the internal exposure at the tissue level in response to a given external exposure (Wells et al., 2004), such as compressive and A-P shear loads. With the given task constraints of lifting a fixed mass load from the ground to waist height in this experimental design it is possible that the degrees of kinematic freedom allowed were not sufficient to influence the magnitude of external loads on the low back, whereas lifters did have the ability to maintain a more upright trunk and leveraging their lower body in the occupation-specific lifts as a protective mechanism to better tolerate low back loads. While data collected in this study cannot be used to directly investigate this potential, future work is recommended to determine whether theoretical experts maintaining more upright trunk postures is an MSD prevention strategy or related to other objectives such as maintaining line of sight with a partner as anecdotally noted.

Identification of expertise level differences in lifting strategy in job-task specific lifts (i.e., backboard and stretcher) but not in generic lifts (i.e., barbell and crate) is novel and important. Based on this finding, it seems that the role of expertise on lifting strategy is relevant to the context in which the expertise was gained. The importance of context in shaping lifting strategy aligns with the conceptual dynamical systems framework on movement (Glazier, 2017; Newell, 1986) where it is hypothesized that the interaction of task (like the implement being lifted in our study), environment and personal constraints (like expertise as considered in our study) influence the control and coordination of movement. This interpretation has practical implications where lift training programs that present generalized presentation of knowledge on lifting mechanics or provides technique training not specific to job demands are unlikely to result in retained and transferrable changes in lifting behavior to reduce low back loads. Additionally, greater intervention beyond providing workers a chance to practice job-relevant lifts is likely needed to reduce the low back loads experienced in practice where the kinematic differences observed in the contextual expert groups did not translate to reductions in these loading exposures. Taken together these data provide experimental support for practical recommendations to improve lift training provide by Denis et al. (2020) by supporting that complimenting theoretical knowledge with motor engagement (i.e., actively practicing lifting) and using real working situations may be necessary to lead to retained behavior changes that do not necessarily reduce loads, but improve one’s tolerance to those loads. Appropriately dosed physical exercise (especially resistance training) may also be considered as part of a comprehensive physical preparation plan for MSD prevention and management (Sowah et al., 2018; Sundstrup et al., 2020).

This study offers insights on the effect of expertise on lifting strategy, but it is not without limitation. First, although care was taken to differentiate between novices, contextual experts, and theoretical experts there was still heterogeneity in these sample groups. Of note, individuals across the expertise groups had varying levels strength and conditioning experience. While strength and conditioning experience was controlled for in the novice group, across the contextual and theoretical expert groups there were examples of individuals who frequently participated in lifting within a gym environment which could have influenced their lifting strategy in the barbell and/or crate lifting tasks. Having an extensive strength and conditioning background is likely consistent with deliberate practice to improve lifting strategy, which could have confounded the results of this study. Indeed, there is preliminary evidence that efforts to focus on exercise technique during training may influence how movements are coordinated and controlled when executing unrehearsed occupational tasks (Frost, Beach, Callaghan, & McGill, 2015). Unfortunately, the varying levels of recreational lifting experience was not controlled for in the study design and so should be considered in the interpretation of findings. Second, the mass and environment present in the occupation-specific lifting tasks did not directly replicate the paramedic work environment. This is an important consideration as load mass has been shown to influence resultant lifting strategy (Albert et al., 2008; Sadler et al., 2011; Sheppard et al., 2016). However, the load mass used in this study was selected to be within the reported mean patient mass of 65.3–81.9 kg (Coffey et al., 2016), but lower within the range to recruit a diverse sample including novice lifters with potentially lower underlying strength. Finally, the controlled lab environment did not replicate the dynamic and potentially time-sensitive environment that may be present when paramedics are responding to emergency calls. The difference in environment could influence resulting lifting strategy (Newell, 1986) and is therefore an important consideration moving forward when extrapolating these study findings to consider how paramedics lift on the job.

In this study we investigated whether type of expertise is a determinant of lifting strategy in either occupation-specific or generalized lifting tasks. Expertise did not influence the magnitude of low back loads experienced when lifting, providing evidence that expertise (as described in this study) may not be a causative factor that influences individuals to define a movement objective to minimize peak resultant low back loads when performing occupational lifts. However, expertise did influence lifting strategy in lifts specific to the occupation that the expertise was gained in. Experts may be choosing lifting strategies specific to the context of those lifting tasks, like preserving line-of-sight for communication with a lifting partner. Alternatively, they may be lifting in a way to enhance their ability to tolerate the exposures associated with lifting. Training novices to move like experts is unlikely to result in reduced low back loads during lifting but may serve to enhance novice’s tolerance to withstand those exposures, but specific to contextually relevant lifting tasks.

## Key Points

- Peak compression and anteroposterior shear low back loads in lifting did not differ as a function of expertise
- Contextual experts exhibited differences in lifting kinematics, but only in occupation-specific lifts
- Modeling the lifting strategy of experts may help novices adopt lifting strategies that enable them to better tolerate low back loads during contextually relevant lifting
